# Cryo-electron Microscopy Structure and Transport Mechanism of a Wall Teichoic Acid ABC Transporter

**DOI:** 10.1128/mBio.02749-19

**Published:** 2020-03-17

**Authors:** Li Chen, Wen-Tao Hou, Tao Fan, Banghui Liu, Ting Pan, Yu-Hui Li, Yong-Liang Jiang, Wen Wen, Zhi-Peng Chen, Linfeng Sun, Cong-Zhao Zhou, Yuxing Chen

**Affiliations:** a Hefei National Laboratory for Physical Sciences at the Microscale and School of Life Sciences, University of Science and Technology of China, Hefei, Anhui, China; b School of Chemistry and Materials Science, University of Science and Technology of China, Hefei, Anhui, China; c CAS Centre for Excellence in Molecular Cell Science, University of Science and Technology of China, Hefei, Anhui, China; University of Guelph; Fred Hutchinson Cancer Research Center

**Keywords:** cryo-EM, *Staphylococcus aureus*, inhibitors, wall teichoic acids, ABC transporters, MRSA, structure

## Abstract

The wall teichoic acid (WTA) is a major cell wall component of Gram-positive bacteria, such as methicillin-resistant Staphylococcus aureus (MRSA), a common cause of fatal clinical infections in humans. Thus, the indispensable ABC transporter TarGH, which flips WTA from cytoplasm to extracellular space, becomes a promising target of anti-MRSA drugs. Here, we report the 3.9-Å cryo-electron microscopy (cryo-EM) structure of a 50% sequence-identical homolog of TarGH from Alicyclobacillus herbarius at an ATP-free and inward-facing conformation. Structural analysis combined with activity assays enables us to clearly decode the binding site and inhibitory mechanism of the anti-MRSA inhibitor Targocil, which targets TarGH. Moreover, we propose a “crankshaft conrod” mechanism utilized by TarGH, which can be applied to similar ABC transporters that translocate a rather big substrate through relatively subtle conformational changes. These findings provide a structural basis for the rational design and optimization of antibiotics against MRSA.

## Abstract (precis)

The wall teichoic acid (WTA) is a major component of cell wall and a pathogenic factor in methicillin-resistant Staphylococcus aureus (MRSA). The ABC transporter TarGH is indispensable for flipping WTA precursor from cytoplasm to the extracellular space, thus making it a promising drug target for anti-MRSA agents. The 3.9-Å cryo-EM structure of a TarGH homolog helps us to decode the binding site and inhibitory mechanism of a recently reported inhibitor, Targocil, and provides a structural platform for rational design and optimization of potential antibiotics. Moreover, we propose a “crankshaft conrod” mechanism to explain how a big substrate is translocated through subtle conformational changes of type II exporters. These findings advance our understanding of anti-MRSA drug design and ABC transporters.

## INTRODUCTION

Methicillin-resistant Staphylococcus aureus (MRSA) is a common clinical pathogen leading to difficult-to-treat, in many cases even fatal, infections in humans (1). Due to the abuse of antibiotics in recent decades, many MRSA strains are resistant to all β-lactams, even to the last-resort antibiotic vancomycin (2). Most of the present clinical β-lactam antibiotics target the peptidoglycan synthesis pathway (3). However, the emergence of various MRSA strains makes an urgent appeal to develop new antibiotics against enzymes controlling the biosynthesis of other cell wall components, such as wall teichoic acids and lipoteichoic acids (WTAs and LTAs, respectively).

WTAs are anionic glycopolymers attaching to the peptidoglycan of Gram-positive bacteria (4) and are the key components that comprise up to 50% of the total mass of cell wall (5, 6). WTAs are crucial for cell division, biofilm formation, host colonization, and infection (1, 7). Some S. aureus strains have evolved a mechanism to escape hosts’ immune systems via modifying their WTAs into altered structures that resist host lysozyme or corresponding antibodies (8, 9). Thus, the enzymes that control the rate-limiting steps of the WTA biosynthesis pathway become a pool of candidates for developing novel antibiotics against MRSA infections (10).

Undecaprenyl diphosphate (UND-PP) is a key lipid carrier for the biosynthesis of WTAs and a variety of other cell wall polysaccharide components, such as lipopolysaccharides (11), bacterial peptidoglycan (12), and capsular polysaccharides (13). However, linking to UND-PP is not sufficient *per se* for the sugar chain to pass the thermodynamic barrier of a lipid bilayer via free diffusion (14). Therefore, a group of dedicated flippases in the ATP-binding cassette (ABC) superfamily have been evolved. In S. aureus, ABC transporter TarGH is indispensable for the transmembrane flipping of WTA precursor, an *N*-acetylglucosamine (GlcNAc)-modified ribitol-phosphate (RboP) polymer covalently linked to the UND-PP moiety, out of the cell membrane (1, 10) (see Fig. S1 in the supplemental material). Aiming at TarGH as a potential target, a well-known inhibitor, Targocil, with a MIC of 0.3 μM, was recently identified by Lee and coworkers (15). Moreover, random screening of the anti-Targocil S. aureus strains has identified a couple of putative Targocil binding residues on TarGH (16). However, the biochemical and structural evidence is absent.

10.1128/mBio.02749-19.1 FIG S1 Schematic of the primary Staphylococcus aureus WTA biosynthetic pathway. The scheme displays the intercellular pathway of WTA biosynthesis by several key enzymes and finally the poly(ribitol-phosphate) polymer is transported to the outside by TarGH, after which WTA is covalently linked through a phosphodiester bond to the MurNAc sugars of peptidoglycan by an unidentified enzyme. Download FIG S1, TIF file, 2.9 MB. Copyright © 2020 Chen et al. 2020 Chen et al. This content is distributed under the terms of the Creative Commons Attribution 4.0 International license.

The present structural knowledge on the ABC flippases that transport diverse UND-PP-linked substrates is limited to two structures. One is Pglk from human pathogen Campylobacter jejuni, which translocates intracellular UND-PP-GlcGalNAc_5_Bac out of the membrane for the N-glycosylation of bacterial surface proteins (17). The structure depicted a type I exporter that adopts a lateral access and “outward only” mechanism (18). Another is a type II exporter from Aquifex aeolicus (19, 20), termed Wzm-Wzt, the homolog of which in Escherichia coli O9a flips a UND-PP-linked intermediate into the periplasm for the synthesis of O antigens (11). Notably, the extra carbohydrate binding domain, which plays an important role in recognizing the sugar moiety of the substrate (21, 22), was deleted in the crystal structure, termed Wzm-WtzN. However, the structural and functional diversity of UND-PP-linked substrates make the transport mechanism of these ABC flippases poorly understood. Here, we determined the cryo-electron microscopy (cryo-EM) structure of Alicyclobacillus herbarius TarGH at 3.9 Å, which allowed us to gain more insights into the WTA transport driven by S. aureus TarGH and provided a structural platform for the rational design and further optimization of inhibitors of WTA flipping.

## RESULTS

### Overall structure of TarGH.

**FIG 1:**
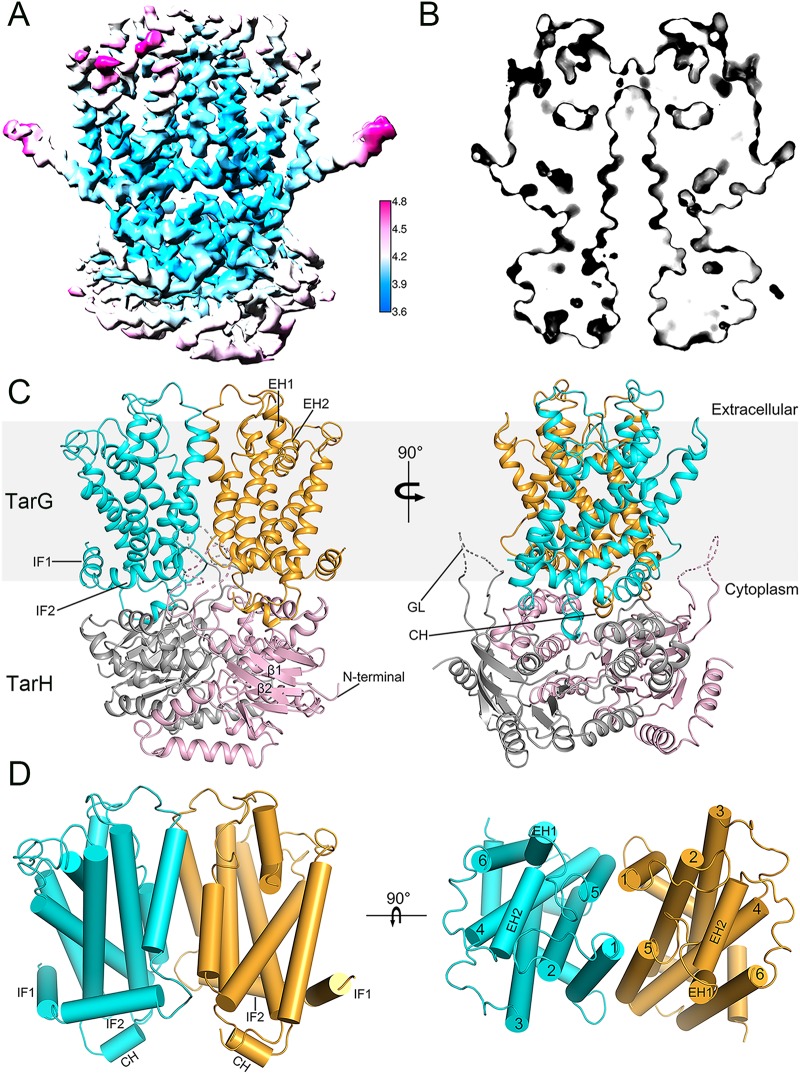
Structure of TarGH (TarH_E169Q_) from Alicyclobacillus herbarius. (A) The local resolution map calculated using Relion. The overall resolution for the EM map is about 3.9 Å, while the local resolution for the core region reaches 3.6 Å. (B) TarGH is in an inward-facing conformation. A section view for the protein surface is shown here, indicating that the tunnel is closed at the extracellular side and accessible from the cytoplasm. (C) Overall structure of TarGH. TMDs encoded by *tarG* are shown in cyan and orange, and NBDs encoded by *tarH* are shown in gray and pink. (D) Structure of TMDs. The gate loop (GL), coupling helices (CHs), extracellular gate helices (EH1 and EH2), and interface helices (IF1 and IF2) are labeled. The TM helices are numbered sequentially.

After systematic trials, we failed in overexpressing S. aureus TarGH; however, we succeeded in purifying the recombinant TarGH homolog from *A. herbarius* (see Fig. S3A in the supplemental material). As it shares an overall sequence identity of ∼50%, it should be an ideal prototype of S. aureus TarGH (Fig. S2A and B). Using the cryo-electron microscopy (cryo-EM) technique, we solved the structure of *A. herbarius* TarGH at an overall resolution of 3.9 Å (Fig. 1A), with the catalytic residue Glu169 mutated to Gln. Analysis of the regional resolution showed that the core structure has a relatively high resolution of 3.6 Å, whereas the surface-exposed regions possess a rather lower resolution. In general, the nucleotide-binding domains (NBDs), which are encoded by the *tarH* gene, have a relatively lower resolution than the transmembrane domains (TMDs) encoded by the *tarG* gene.

10.1128/mBio.02749-19.2 FIG S2 Sequence alignment of wall teichoic acid transporters. (A) Alignment of TMDs between Alicyclobacillus herbarius and Staphylococcus aureus. (B) Alignment of NBDs between Alicyclobacillus herbarius and Staphylococcus aureus. (C) Alignment of TMDs from different strains. Download FIG S2, TIF file, 2.8 MB. Copyright © 2020 Chen et al. 2020 Chen et al. This content is distributed under the terms of the Creative Commons Attribution 4.0 International license.

10.1128/mBio.02749-19.3 FIG S3 Cryo-EM analysis of TarGH (TarH_E169Q_). (A) A representative gel filtration analysis of TarGH (TarH_E169Q_) in UDM. The second peak fraction is visualized on SDS-PAGE gels by Coomassie blue staining. (B) A representative cryo-EM micrograph. Some particles are circled by red circles. (C) Euler angle distribution of the final 3D refinement of the overall map. (D) Representative reference-free 2D class averages of the TarGH (TarH_E169Q_) particles. (E) The gold-standard Fourier shell correlation (FSC) curves for the overall map. (F) FSC curves of the refined model versus the overall 3.94-Å map against which it was refined (black), of the model refined in the first of the two independent maps used for the gold-standard FSC versus that same map (green), and of the model refined in the first of the two independent maps versus the second independent map (red). The small difference between the red and green curves indicates that the refinement of the atomic coordinates does not suffer from overfitting. Download FIG S3, TIF file, 2.8 MB. Copyright © 2020 Chen et al. 2020 Chen et al. This content is distributed under the terms of the Creative Commons Attribution 4.0 International license.

In the absence of ATP, TarGH adopts an inward-open conformation (Fig. 1B). TarH/NBD possesses a classic NBD fold of ABC transporter, with an ɑ-helical subdomain and a RecA-like ATPase core subdomain. All conserved motifs, including Walker A and B, the ABC signature motif, could be clearly assigned in our structure. The distance between the hydroxyl groups of Ser64 on Walker A from one NBD and Ser146 on the signature motif from the other is about 6 Å, suggesting a resting state of TarGH ready for ATP binding.

Similar to other type II ABC exporters (23), the TMDs of TarGH consist of 6 + 6 transmembrane helices (TMs) without domain swapping (Fig. 1C and D). TM1 of one TMD packs against TM5 of the other, and vice versa, forming a total interface area of 1,110 Å^2^. We also found two reentrant helices between TM5 and TM6 that insert in the outer leaflet of membrane, termed extracellular helices EH1 and EH2 (Fig. 1C), like other type II exporters (19, 23, 24). In addition, each TMD is preceded by an extra amphipathic helix at the N terminus, compared to that in Wzm-WztN (19), sticking at the interface between the membrane and cytosol; thus, these are termed the interface helices IF1 and IF2 (Fig. 1C). Notably, this extra IF1 is conserved in most teichoic acid transporters (Fig. S2C). As in a typical ABC transporter, the conformational changes between TMDs and NBDs are also interconnected by the coupling helix (CHs), a short helix between TM2 and TM3. Each CH protrudes from the TMD and is embedded in a groove on the NBD at the same side without domain swapping. In addition, a flexible loop between β1 and β2 of the NBD extends toward the TMD, forming a putative substrate entrance on each side (Fig. 1C). Remarkably, the distal moiety of the corresponding loop in the Wzm-WztN structure, which is folded into a short α-helix due to direct contacts with the TMD, was proposed to function as a gate of the substrate binding pocket and termed the gate helix (GH) (19). Accordingly, we propose that the flexible loop in our structure functions as a gate loop (GL).

### The putative substrate translocation tunnels.

**FIG 2:**
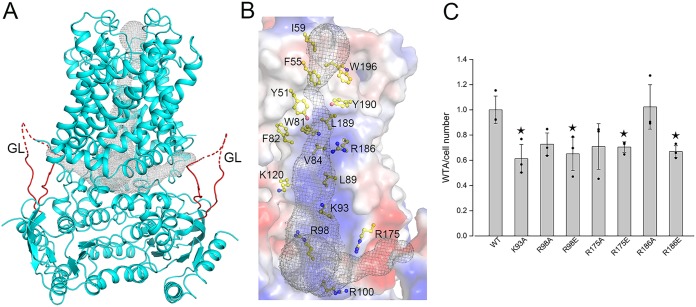
The putative substrate tunnel. (A) Two L-shaped tunnels were simulated using the program Caver 3.0.1, as indicated by gray mesh. GLs close to the entrance are shown in red. (B) Hydrophobic residues surrounding the vertical path and positively charged residues located at the turn of the entrance are shown as sticks. (C) Quantification of WTA in S. aureus NCTC8325 transformed with pLI50-*tarG* and its mutants. At least three independent experiments were performed for each assay. The means and standard deviations were calculated, and the data are presented as means ± standard deviations. Two results of three experiments in the wild-type (WT) group were overlapped due to similar values. Two-tailed Student’s *t* test is used for the comparison of statistical significance. The *P* values <0.05, 0.01, and 0.001 are indicated with single, double, and triple stars, respectively.

Using the program Caver 3.0.1 (25), with the probe radius set to 1.1 Å, we simulated two back-to-back L-shaped substrate tunnels, each of which has a horizontal entrance along the membrane-cytosol interface and a vertical path through the cell membrane (Fig. 2A). The entrance starts at a putative gate, which is guarded by three loops, namely, GL, Loop_IF2-TM1_, and Loop_TM4-TM5_, and extends to the sharp turn of the L-shaped tunnel (Fig. 2A). The turn is surrounded by a line of positively charged residues, Lys93, Arg98, Arg100, Lys120, Arg175, and Arg186, whereas the transmembrane path is mainly composed of hydrophobic residues, such as Tyr51, Phe55, Trp81, Phe82, Val84, Leu89, Leu189, Tyr190, and Trp196 (Fig. 2B). Notably, this tunnel is complementary in charge to the structure of the UND-PP moiety, which has a lipid chain followed by two acidic phosphate groups (Fig. S6B).

In fact, a similar tunnel with a positively charged entrance followed by a hydrophobic path also exists in the structures of Pglk (18) and Wzm-WztN (19). Transport activity assays proved that UND-PP-linked polysaccharides of various lengths could be recognized and exported by Pglk (18). In addition, complementary experiments indicated that the Bacillus subtilis *tagGH* gene could be replaced by S. aureus *tarGH*, without significant difference in the synthesis of WTA (16). It suggested that the UND-PP moiety, rather than the polysaccharide chain, determines the recognition and initiation of the entry of full-length substrate (16, 18, 26). We proposed that at the initial step of substrate binding, the diphosphate group of the UND-PP moiety guides the substrate binding to TarGH via specific interactions with the positively charged residues at the turn of the tunnel entrance. Notably, only a part of residues along the GL could be assigned to the electron density map in our structure (Fig. S5B), indicating its high flexibility and weak interactions with the neighboring segments of TMDs.

To ensure the *in vivo* role of these positively charged residues for the transport of WTA, we employed the so-called dominant negative method (27) to introduce mutant *tarG* into the strain S. aureus NCTC8325. Briefly, the wild-type and mutant *tarG* genes, both of which possess the native promoter of *tarG* and a region coding for the Flag tag at the N terminus, were introduced in S. aureus NCTC8325 using the shuttle plasmid pLI50. Western blotting against the antibody to Flag tag indicated the expression of the introduced TarG (Fig. S6C). The expressed TarG (both wild type and mutant) encoded by the plasmid could compete the original one encoded by the genome during the formation of ABC transporter TarGH. Single mutation of any of the four conserved positively charged residues, namely, Lys93, Arg98, Arg175, and Arg186, into either alanine or glutamate led to decrease of the WTA quantity at about 30% to 40%, compared to the wild type (Fig. 2C). Notably, we failed in obtaining the K93E mutant transformant, indicating that this mutation is most likely lethal to S. aureus. After data analysis, we found the mutation to glutamate generally displayed a more significant decrease of WTA yield than the corresponding mutation to alanine, which indicates the importance of the electrical property of these residues in the tunnel.

Scanning along the vertical path, we found a constrained bottleneck at the tunnel exit toward the extracellular space (Fig. 2B), which is guarded by Tyr51, Phe55, and Tyr190 from each TMD. The bottleneck has a radius of about 1.1 Å, which is unlikely to allow the water molecule to diffuse across the membrane through the transporter. It revealed that the present inward-open structure of TarGH possesses a closed exit of the substrate tunnel. Multiple-sequence alignment of TarG and homologs revealed that these three hydrophobic residues are highly conserved (Fig. S2C), indicating that these flippases might share a conserved bottleneck close to the substrate tunnel exit. Furthermore, we speculated that at the resting state, this bottleneck may prevent the diffusion of small molecules through the tunnel.

### Mapping the Targocil binding site on the TarGH structure.

**FIG 3:**
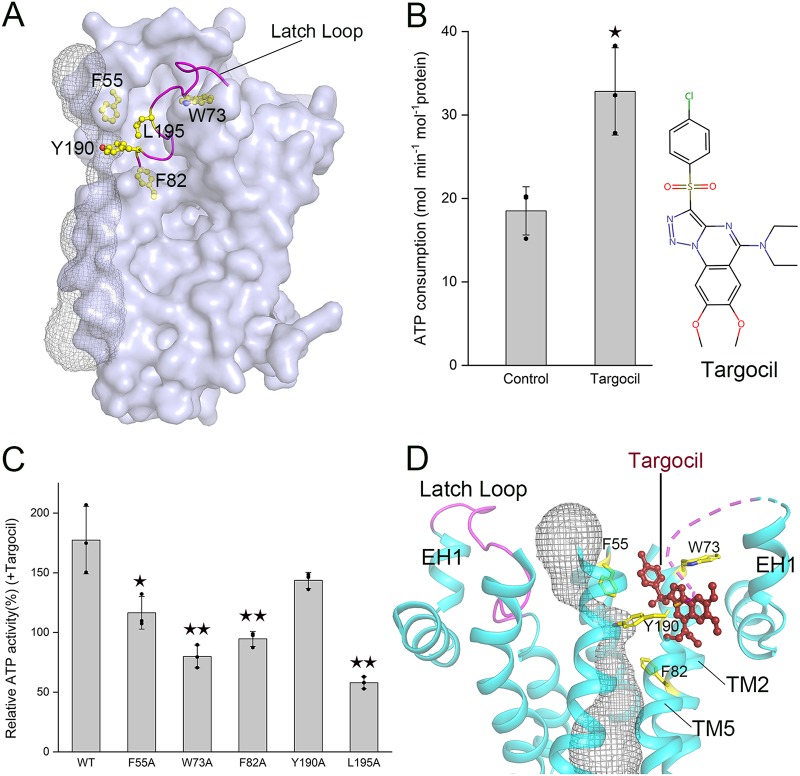
The binding site of Targocil. (A) Five conserved Targocil-resistant residues (F55, W73, F82, Y190, and L195) are shown as yellow sticks, and the latch loop is shown in magenta. (B) ATPase activity of wild-type TarGH in the absence or presence of Targocil. (C) Relative ATPase activity of wild-type (WT) TarGH and mutants in the presence of Targocil. At least three independent experiments were performed for each assay of panels B and C. The means and standard deviations were calculated, and the data are presented as means ± standard deviations. Two results of three experiments in the control group of panel B were overlapped due to similar values. Two-tailed Student’s *t* test is used for the comparison of statistical significance. The *P* values of <0.05, 0.01, and 0.001 are indicated with single, double, and triple stars, respectively. (D) A docking model of Targocil on TarGH. The four Targocil-resistant residues F55, W73, F82, and Y190 flanking the bottleneck of the exit are shown in yellow.

The emergence of MRSA makes it an urgent necessity to develop a novel strategy different from the traditional therapeutic that uses antibiotics only. A recent report suggested that pretreatment with the WTA inhibitors could restore the β-lactam susceptibility of MRSA; thus, combined administration of medicines is capable of significantly reducing drug resistance (28). As a rate-limiting enzyme responsible for the last step of the WTA biosynthesis pathway, S. aureus TarGH was proposed to be a promising target of WTA inhibitors (4, 26). Indeed, a well-known inhibitor, termed Targocil, was chemically synthesized derived from a lead compound against MRSA (15). Moreover, sequence analysis of the Targocil-resistant S. aureus strains identified seven putative residues of TarGH involved in binding to Targocil (16). Thanks to the high sequence identity, five out of these seven Targocil-resistant residues of S. aureus TarGH could be eventually mapped on our present *A. herbarius* TarGH structure (Fig. 3A). Remarkably, four of these residues, Phe55, Trp73, Phe82, and Tyr190, on each TMD form a hydrophobic pocket (Fig. 3A), which is occupied by the loop between TM5 and EH1 that harbors another Targocil-resistant residue, Leu195. In addition, the pocket-forming residues Tyr190 and Phe55 also constitute one side of the bottleneck close to the tunnel exit (Fig. 2B), indicating a direct cross talk between this hydrophobic pocket and the bottleneck.

Hence, we chemically synthesized Targocil, the structure of which was further proved by the nuclear magnetic resonance spectrum (Fig. S6A). According to the previously reported MIC (15), Targocil at a 1 μM final concentration was applied to the ATPase activity assays of TarGH reconstituted in liposomes. In the absence and presence of Targocil, TarGH possesses a *V*_max_ of ATPase activity of 18.5 and 32.8 mol min^−1 ^mol^−1^ protein, respectively (Fig. 3B). A significant increase of ATPase activity upon the addition of Targocil indicated that Targocil indeed specifically binds to TarGH. We speculated that conformational changes induced by the inhibitor may facilitate TarGH to adopt a conformation with elevated ATPase activity, in agreement with a recent report on human ABCB1, which gains an enhanced ATPase activity upon the addition of inhibitors (29).

To further precisely determine the binding site of Targocil, we sequentially mutated the five residues which were previously identified in S. aureus TarGH and conserved in *A. herbarius* TarGH. As predicted, except for Y190A, single mutation of any one of the other four Targocil-resistant residues resulted in a much less significant change of TarGH ATPase activity upon the addition of Targocil (Fig. 3C). These results not only confirmed the previously identified Targocil-resistant residues (16) but also provided the direct biochemical and structural evidence that these residues are involved in Targocil binding.

Based on our ATPase activity assays in the presence of Targocil, we can constrain the possible binding site within in a limited space. Notably, Targocil and the loop between TM5 and EH1 most likely exclusively bind to the hydrophobic pocket composed of Targocil-resistant residues; thus, we suspected that it might be shifted out of the pocket upon Targocil binding, and we termed it the latch loop. Indeed, once the latch loop was removed, Targocil could be well docked to the empty pocket using AutoDock Vina (30), at a lowest binding affinity energy of −8.1 kcal mol^−1^ (Fig. 3D and Fig. S6D). Notably, two of the binding residues in our docking model, Phe55 and Tyr190, in addition to Leu195 on the latch loop (Fig. 3A), are exactly flanking one side of the bottleneck (Fig. 3D). It suggested that the tunnel exit is most likely restrained upon the inhibitor binding, leading to a decreased yield of WTA. In other words, the latch loop, which mimics the inhibitor, is also involved in the regulation of the opening and closing of the bottleneck. The results of ATPase activity assays in combination with the docking model enabled us to propose that Targocil serves as a wedge, which makes EH1, EH2, and TM6 shift outward, accompanying the inward shift of TM1 and TM5. Consequently, the tunnel becomes narrower, and meanwhile, the TMDs are slightly enlarged at the outward face, corresponding to the approach of the NBDs toward each other and a higher ATPase activity, in agreement with our results from activity assays (Movie S1).

10.1128/mBio.02749-19.8 MOVIE S1 Mechanism of Targocil stimulation of ATPase activity. The TMD, NBD, coupling helix, and hydrophobic pocket in TMD and Targocil are shown in the movie by the blue quadrilateral, red circle, yellow polygon, and black polygon, respectively. When TarGH binds Targocil, Targocil inserts into the hydrophobic pocket constituted by Targocil-resistant amino acids in TMD like a wedge, which makes the EH1, EH2, and TM6 (outside TarGH) shift outward, along with the inward shift of TM1 and TM5 (inside TarGH). Consequently, the tunnel becomes narrower, and meanwhile the TMDs are slightly enlarged at the outward face, corresponding to the approach of the NBDs toward each other and a higher ATPase activity. Download Movie S1, AVI file, 0.9 MB. Copyright © 2020 Chen et al. 2020 Chen et al. This content is distributed under the terms of the Creative Commons Attribution 4.0 International license.

### Structural comparison revealed a “crankshaft conrod” mechanism that amplifies the subtle conformational changes of TMDs.

**FIG 4:**
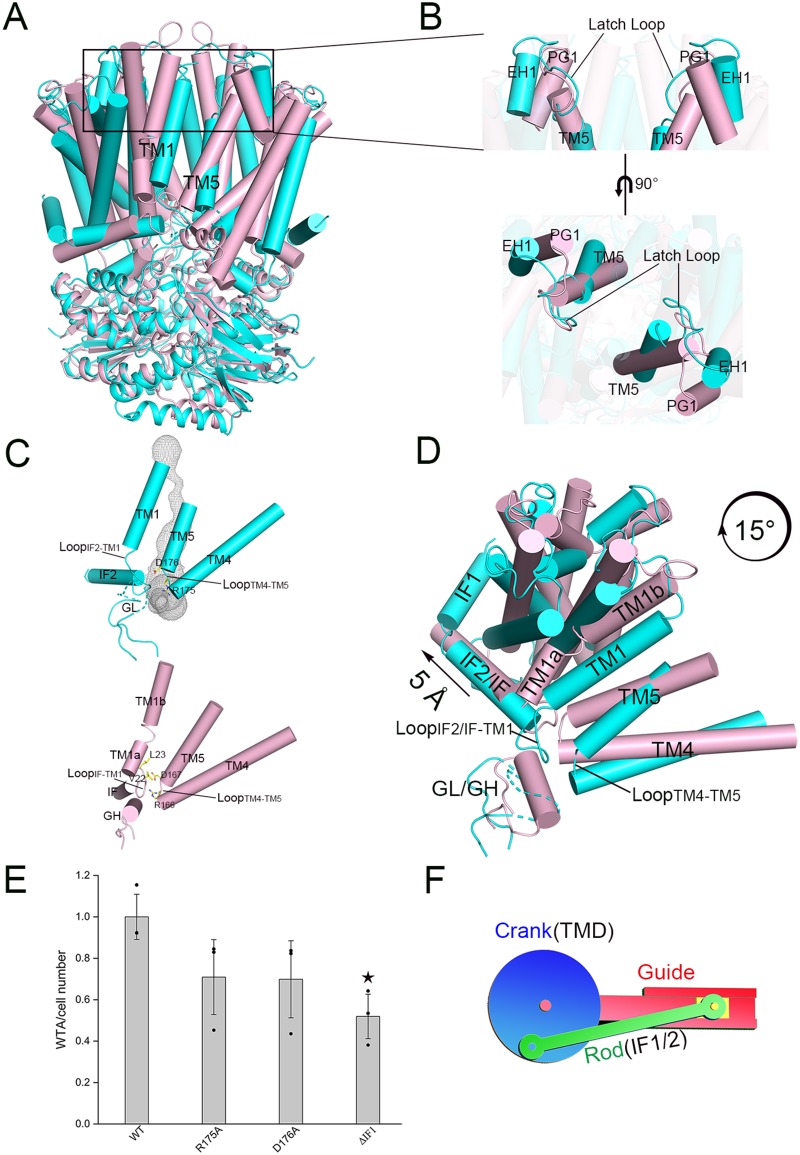
Structural comparison. (A) Overall structure superposition of ATP-free TarGH on ATP-bound Wzm-WztN. (B) A close look at the latch loop region. (C) The entrance of the substrate tunnel in ATP-free TarGH and ATP-bound Wzm-WztN. (D) A top view of superimposed TMDs of ATP-free TarGH and ATP-bound Wzm-WztN. (E) Quantification of WTA in S. aureus NCTC8325 transformed with pLI50-*tarG* and its D-R motif mutants, as well as the IF1 truncation (ΔIF1) version. At least three independent assays were performed. At least three independent experiments were performed for each assay. The means and standard deviations were calculated, and the data are presented as means ± standard deviations. Two results of three experiments in the wild-type (WT) group were overlapped due to similar values. Two-tailed Student’s *t* test is used for the comparison of statistical significance. The *P* values of <0.05, 0.01, and 0.001 are indicated with single, double, and triple stars, respectively. (F) A scheme of the crankshaft conrod and corresponding structural components. TarGH is shown in cyan, and Wzm-WztN is shown in pink. The corresponding structural segments including TM1, TM5, the latch loop, GL/GH, Loop_IF-TM1_, and Loop_TM4-TM5_ are labeled.

Structural superposition using PyMol (https://pymol.org/) by the centers of molecules of our ATP-free TarGH against the Wzm-WztN structures in the ATP-free and ATP-bound forms gave a root mean square deviation (RMSD) of 4.7 Å over 832 Cα atoms and 3.4 Å over 595 Cα atoms, respectively. In addition, superposition of the TMDs yielded an RMSD of 3.9 Å over 375 Cα atoms and 3.4 Å over 341 Cα atoms, respectively. It suggested that our structure resembles the two structures of Wzm-WztN, which undergo subtle conformational changes upon ATP binding. Moreover, compared to the ATP-bound Wzm-WztN, fewer conformational changes could be observed in the overall structure of our ATP-free TarGH, except for a rigid-body rotation of the TMDs at about 15° (Fig. 4A and E). In contrast to the blocked tunnel at the bottleneck in our ATP-free structure, a pair of TM5 helices that constitute the wall of the substrate tunnel tilt away from each other, along with the corresponding latch loops flipping outward, resulting in opening of the tunnel in the structure of Wzm-WztN upon ATP binding (Fig. 4B), which eventually enables the substrate to be pumped out.

Structural comparison revealed that TarGH possesses an open tunnel entrance in the absence of ATP (Fig. 4C), compared to a closed entrance in the structure of ATP-bound Wzm-WztN (Fig. 4C). Due to the high flexibility of the gate loop (GL), the entrance of the substrate tunnel in TarGH is accessible. In contrast, the entrance in the structure of ATP-bound Wzm-WztN is closed due to the conformational changes at two loops, Loop_IF-TM1_ and Loop_TM4-TM5_, in addition to the GH, which is folded most likely via interactions with the adjacent loops. A couple of interactions among the three segments were observed in ATP-bound Wzm-WztN (Fig. 4C), including the hydrogen bond of Gln18 from GH and Thr21 from Loop_IF-TM1_, in addition to hydrophobic and nonbonded contacts among the three segments, resulting in a closed entrance. Sequence analysis indicated that the GL possesses positively charged residues, such as Lys18, Arg19, and Arg23 in TarGH, and Loop_TM4-TM5_ contains a very conserved Arg175-Asp176 motif (Fig. 4C and Fig. S2C). In addition, the conserved aspartate residue was proposed to be responsible for the binding of the diphosphate moiety of UND-PP (31, 32). Similarly, a substrate tunnel entrance clustering of positively charged residues in addition to a conserved aspartate residue, Asp167, could be found in Wzm-WztN (Fig. 4C). In fact, the WTA yield assays using the dominant negative method revealed that mutation of either Arg175 or Asp176 to alanine somewhat impairs the WTA synthesis, leading to an ∼30% decrease of total WTA (Fig. 4E).

Despite the subtle changes of overall structure, along with the clockwise rotation of the TMD of ATP-bound Wzm-WztN against that of TarGH, the interface helix IF that sticks on the TMD undergoes a horizontal translation of about 5 Å. In consequence, the loop succeeding the IF, namely, Loop_IF-TM1_, shifts the tunnel entrance inward, along with the approach of GH toward Loop_TM4-TM5_ (Fig. 4D). It suggested that the subtle conformational changes of TMDs could be transmitted and amplified by the IF, which triggers a rather significant conformational change of the tunnel entrance. This transformation of the force is reminiscent of the mechanical device the crankshaft conrod (Movie S2). In the present case, the TMD mimics the crank, whereas the IF resembles the rod (Fig. 4F). To compensate for the fewer conformational changes upon ATP binding, TarGH possesses an extra interface helix IF1 at the N terminus, in addition to IF2, which corresponds to the IF of Wzm-WztN. As predicted, truncation of IF1 resulted in a significant reduction of WTA yield to about 50%, compared to the wild type (Fig. 4E). Altogether, TarGH adopts a crankshaft conrod mechanism to pump out a relatively big substrate through subtle conformational changes along the NBDs and TMDs.

10.1128/mBio.02749-19.9 MOVIE S2 Conformational change of TMD in the “crankshaft conrod” mechanism. This video is produced based on the structure alignment of TarGH (cyan) and Wzm-WztN (ATP bound, PDB accession no. 6M96, pink). From the top view, when TarGH binds to ATP, TMD of TarGH clockwise rotates about 15° as a rigid body. Along with this rotation, the interface helix IF that sticks on the TMD undergoes a horizontal translation of about 5 Å. The corresponding structural segments, including TMD, IF2/IF, and GL/GH, are labeled. Download Movie S2, AVI file, 1.5 MB. Copyright © 2020 Chen et al. 2020 Chen et al. This content is distributed under the terms of the Creative Commons Attribution 4.0 International license.

## DISCUSSION

**FIG 5:**
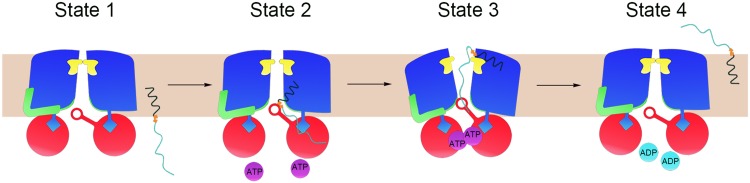
A putative transport cycle. States 1, 2, 3, and 4 represent the resting state, substrate binding state, ATP binding state, and ATP hydrolyzed state after substrate translocation, respectively. The NBD, TMD, latch loop, and IF, and their corresponding loops, are colored in red, blue, yellow, and green, respectively. The coupling helix and gate loop are shown as a blue rectangle and a red circle protruding from the TMD and NBD, respectively. The substrate is shown as a curled line.

The present structure of TarGH, in combination with previous studies (19, 23, 24, 33–35), enabled us to propose a crankshaft conrod-aided transport cycle for type II exporters (Fig. 5). The ATP-free TarGH adopts a resting state, with the substrate tunnel closed at the exit and open at the entrance (Fig. 5, state 1), as shown in our cryo-EM structure. At this state, the latch loop blocks the exit and functions as an autoinhibitor that mimics the exogenous inhibitor Targocil, while the entrance formed by the GL and Loop_TM4-TM5_ is open and ready for substrate recognition and binding. At the initial step, the diphosphate group of the UND-PP moiety could be first recognized by the conserved D-R motif at the entrance, which further guides the approaching substrate (Fig. 5, state 2). Upon binding to ATP, NBDs move toward each other, making TMDs adopt an outward-open conformation (Fig. 5, state 3). In this transition process, the rigid-body rotation of each TMD is transformed to a horizontal translation of the IFs through the crankshaft conrod mechanism. Along with the backward movement of the IFs, the succeeding Loop_IF2-TM1_ bends the TMD inward. In consequence, Loop_TM4-TM5_ and GL are recruited via hydrophobic and hydrophilic interactions to form a closed entrance, which might facilitate the insertion of the sugar moiety to the transmembrane tunnel. However, whether the UND moiety is translocated inside or outside the tunnel remains unknown, although it was previously proposed to stay in the membrane bilayer without entering the TMDs in the cases of Pglk and Wzm-Wzt (18, 20). Meanwhile, latch loops are ejected and the tunnel exit opens toward the extracellular space. At the final step, ATP is hydrolyzed by the dimerized NBDs, the energy of which drives the translocation of the substrate and eventually makes TarGH go back to the resting state (Fig. 5, state 4). As previously proposed (20), the UND moiety most likely continues inserting in the outer leaflet of the membrane whereas the diphosphate group and sugar moiety are exposed to the extracellular space. Notably, it might take several cycles of transport to pump out one molecule of UND-PP-linked oligosaccharide.

In contrast to type I exporters, conformational changes in type II exporters are relatively subtle. How such subtle conformational changes can accomplish the transport of a large substrate remains unknown. Our present cryo-EM structure combined with the previously reported Wzm-WztN crystal structures provided insights into the transformation of the chemical energy of ATP hydrolysis to rigid-body rotation of TMDs, which is further amplified by a crankshaft conrod mechanism to the horizontal translation of the interface helices. This hypothesis can be applied to most ABC exporters possessing similar structural components.

## MATERIALS AND METHODS

### Protein preparation.

The codon-optimized *tarG* and *tarH* genes were synthesized by Sangon Biotech. *tarG* and *tarH* genes were cloned into a single modified pET-19b vector (YouBio) by using the One Step Cloning kit (Vazyme), with the *tarG* product containing an N-terminal decahistidine tag. The plasmid was transformed into the Escherichia coli BL21(DE3) strain (Invitrogen), growing at 37°C in lysogeny broth (LB) culture medium, supplemented with 50 μg ml^−1^ ampicillin. Overexpression of TarGH was induced by adding 0.2 mM IPTG (isopropyl-β-d-thiogalactopyranoside) (Biofroxx) when the cell density reached an optical density at 600 nm (OD_600_) of 0.6 to 0.8. At 4 h after induction at 37°C, cells were collected by centrifugation; homogenized in buffer containing 50 mM Tris-HCl (pH 7.5), 500 mM NaCl, 7 mM β-mercaptoethanol (β-ME) plus 10% glycerol; and stored at −80°C before use.

For purification, cells were thawed followed by disruption with a Constant cell disruption system (Constant Systems) with two passes at 30 kpsi. Cell debris was removed by low-speed centrifugation for 15 min. The supernatant was collected and ultracentrifuged at 200,000 × *g* for 1 h. The membrane fraction was collected and incubated for 1.5 h with buffer containing 50 mM Tris-HCl (pH 7.5), 500 mM NaCl, 7 mM β-ME, 10% glycerol, 1% (wt/vol) dodecyl-β-d-maltopyranoside (DDM; Anatrace), and 1% (wt/vol) octaethylene glycol monododecyl ether (C_12_E_8_; Anatrace) at 4°C. After another round of ultracentrifugation at 200,000 × *g* for 30 min, the supernatant was collected and loaded onto nickel affinity resin (nickel-nitrilotriacetic acid [Ni-NTA]; Qiagen). The column was then washed with 50 mM Tris-HCl (pH 7.5), 500 mM NaCl, 7 mM β-ME, 10% glycerol, and 0.04% DDM. Contaminants were removed by washing with 50 mM Tris (pH 7.5), 500 mM NaCl, 7 mM β-ME, 10% glycerol, and 0.04% DDM plus 20 mM imidazole and 40 mM imidazole successively. Protein was eluted from the affinity resin with buffer containing 50 mM Tris (pH 7.5), 500 mM NaCl, 7 mM β-ME, 10% glycerol, 0.04% DDM, and 400 mM imidazole. Afterward, the eluent was concentrated to 2 ml before further purification by gel filtration (Superdex 200 increase 10/300; GE Healthcare) in the buffer containing 50 mM Tris-HCl (pH 7.5), 500 mM NaCl, 7 mM β-ME, 10% glycerol, and 0.03% *n*-undecyl-β-d-maltopyranoside (UDM; Anatrace). Peak fractions were pooled, and desalting was implemented with buffer containing 20 mM Tris-HCl (pH 7.5), 100 mM NaCl, 7 mM β-ME, 10% glycerol, and 0.03% UDM. Then, it was concentrated for EM analysis.

The mutated plasmid pET19b-*tarGH* (*tarH_E169Q_*) was also constructed by using the One Step Cloning kit (Vazyme). The mutated protein purification was the same as the wild type.

### Sample preparation and cryo-EM data acquisition.

Purified TarGH_E169Q_ was concentrated to 8 mg ml^−1^ for cryo-EM sample preparation implemented with FEI Vitrobot Mark IV. A 3.5-μl amount of protein was applied to a glow-discharged Quantifoil holey carbon grid (1.2/1.3, 300 mesh). Grids were blotted for 4 s with 100% humidity and plunge-frozen in liquid ethane.

Cryo-EM data were collected at liquid nitrogen temperature on a Titan Krios transmission electron microscope (FEI), operating at 300 kV and equipped with a K2 Summit direct electron detector (Gatan) and a Gatan GIF quantum energy filter (slit width, 15 eV). A total of 4,985 micrograph stacks were automatically collected with SerialEM (36) at a nominal magnification of ×59,000 with a defocus range of −1.5 to −2.5 μm, which yielded a final pixel size of 1.36 Å. Each stack was exposed for 8 s with an exposure time of 0.25 s per frame, resulting in 32 frames per stack. The total dose rate was about 64 e^−^/Å^2^ for each stack.

### Image processing.

A simplified flow chart for the image processing procedure is presented in Fig. S4. The motion-corrected, dose-weighted 4,985 micrographs collected from the Institute of Biophysics, Chinese Academy of Sciences, were imported in Relion 2.0 (37). A total of 4,185 micrograph stacks were manually picked for further data processing. To generate a template for automatic particle picking, approximately 2,000 particles were manually picked. A total of 893,306 particles were picked from 4,185 electron micrographs automatically. After two-dimensional (2D) classification, 509,711 particles were selected and subjected to a global angular search three-dimensional (3D) classification. The initial model was generated with images of selected 2D class averages. A total of 305,822 good particles were selected from the local angular search 3D classification and merged together. These particles were then subjected to a local angular search 3D autorefinement, resulting in a 3D reconstruction map with a resolution of 4.18 Å after postprocessing. A guided multireference 3D classification procedure was then applied to the merged data set using Relion 2.0. Particles of the best-classified class were subjected to 3D autorefinement, resulting in a 3D reconstruction map with a resolution of 4.05 Å after postprocessing. A soft overall mask was generated from one of the two unfiltered half-reconstruction maps with a suitable initial binarization threshold using Relion 2.0. The map quality was improved when the particles were subjected to 3D autorefinement with the soft overall mask applied, and the resolution of the reconstruction map after postprocessing reached 3.94 Å. The resolution was estimated with the gold-standard FSC 0.143 criterion (38) with a high-resolution noise substitution method. Local resolution variations were estimated using ResMap (39).

10.1128/mBio.02749-19.4 FIG S4 Cryo-EM data processing. A flow chart for cryo-EM data processing. Details can be found in under “Image processing” in Materials and Methods. Download FIG S4, TIF file, 2.7 MB. Copyright © 2020 Chen et al. 2020 Chen et al. This content is distributed under the terms of the Creative Commons Attribution 4.0 International license.

10.1128/mBio.02749-19.5 FIG S5 EM maps for representative segments of TarGH (TarH_E169Q_). (A) EM maps for IF helices, Latch Loop and each TM helix in the TMD. (B) EM maps for the overall NBD. The gate loop (GL) is shown inside a red rectangle. (C) EM maps for representative segments in the NBD. Download FIG S5, TIF file, 1.8 MB. Copyright © 2020 Chen et al. 2020 Chen et al. This content is distributed under the terms of the Creative Commons Attribution 4.0 International license.

### Model building and refinement.

The overall TarGH map at 3.9 Å was used for *de novo* model building. An all-alanine model was first built manually in Coot (40). Based on the structural alignment between TarGH and homolog Wzm/WztN (PDB code 6OIH), the main TMs were built by homology modeling. The side chains were assigned mainly by bulky residues such as Arg, Phe, Tyr, and Trp. High homology existed between WztN and TarH, so the structure of the WztN of Wzm/WztN was docked into the map and manually adjusted in Coot.

The model was further improved by iterative refinement in real space with secondary structure and geometry restraints using Phenix (41), rebuilt in Coot, and evaluated by MolProbity (42, 43). The structure figures were prepared using the program CHIMERA (44) or PyMOL. Statistics on the 3D reconstruction and model refinement can be found in Table S1.

10.1128/mBio.02749-19.7 TABLE S1 Cryo-EM data collection, refinement, and validation statistics. Download Table S1, DOCX file, 0.02 MB. Copyright © 2020 Chen et al. 2020 Chen et al. This content is distributed under the terms of the Creative Commons Attribution 4.0 International license.

### ATPase activity assays.

All ATP hydrolysis assays were performed in the proteoliposomes. E. coli polar lipids (Avanti) were resuspended in 20 mM HEPES-KOH (pH 7.0) to the final concentration of 20 mg ml^−1^. A 0.45% final concentration of Triton X-100 was added to destabilize the liposomes for 0.5 h at room temperature. The proteins were added to the destabilized liposomes and incubated for 1 h at 4°C. The ratio of lipids and proteins was kept at 100:1 (wt/wt). Triton X-100 was removed by SM-2 adsorbent biobeads (Bio-Rad) by incubation at 4°C overnight and repeated for another 2 h. Proteoliposomes were diluted and resuspended twice with the ice-cold buffer at 250,000 × *g* for 1 h at 4°C. After the final centrifugation, the pellets were resuspended in 20 mM HEPES-KOH (pH 7.0), 50 mM KCl, and 2 mM MgCl_2_ to 100 μl as one reaction sample. For each reaction sample, 0.1 μM TarGH was added.

Targocil was first solubilized in dimethyl sulfoxide (DMSO) to 10 mM and then diluted to 100 μM with double-distilled water (ddH_2_O). For each 100-μl reaction sample, 1 μM Targocil was added, indicating that 0.01% DMSO was included. Thus, we added equal amounts of DMSO as supplements for control groups. Afterward, ATP was added to each sample at a final concentration of 2 mM by three rounds of freezing in liquid nitrogen followed by thawing in a bath sonicator. Reactions were performed at 37°C for 1 h, and the amount of released phosphate ion was quantitatively measured using the ATPase colorimetric assay kit (Innova Biosciences) in 96-well plates at OD_650_.

### Targocil docking on TarGH.

To dock Targocil on our TarGH structure, the latch loop was removed for one symmetry unit by editing the PDB file, as the latch loop has occupied the putative pocket where Targocil most likely binds, based on the previously reported mutation screening and our biochemical assays. Targocil was set as “ligand,” and the edited PDB file was set as “receptor.” We determined the coordinate of the docking space in AutoDock Vina as (128.435, 140.345, 110.712), and the size of the space for the docking was restrained as a 16.0- by 28.0- by 18.0-Å^3^ box. The “exhaustiveness” for the calculation was set to 1,000. The output of the docking produced 9 models, with an affinity from −7.5 to −8.1 kcal/mol. The model with the lowest binding affinity energy is shown in Fig. 3D, and all nine models are displayed with different colors for one symmetric unit in Fig. S6D.

10.1128/mBio.02749-19.6 FIG S6 (A) Nuclear magnetic resonance data for Targocil. (B) Structural formula of undecaprenyl diphosphate. (C) Western blot assay for verifying the expression of S. aureus pLI50-*tarG* (lane 1). (D) Targocil docking models. All nine models calculated by AutoDock Vina are displayed in different colors for one symmetric unit. Download FIG S6, TIF file, 2.3 MB. Copyright © 2020 Chen et al. 2020 Chen et al. This content is distributed under the terms of the Creative Commons Attribution 4.0 International license.

### Dominant negative method to introduce mutant TarGH into S. aureus NCTC8325.

The gene fragment encompassing *tarG* and its native promoter were amplified from S. aureus NCTC8325 genomic DNA. The fragment was cloned into the shuttle plasmid pLI50, and a Flag tag was added at the N-terminal part of the gene product to derive the plasmid pLI50-*tarG*. This plasmid pLI50 with the N-terminal Flag tag was constructed by using the One Step Cloning kit (Vazyme). The mutated pLI50-*tarG* mentioned in this paper was constructed by using the One Step Cloning kit (Vazyme). The wild-type *tarG* and *tarG* mutant plasmids were transformed by electroporation into S. aureus strain RN4220 for modification and subsequently into S. aureus NCTC8325.

### Purification of the recombinant expressed TarG from S. aureus NCTC8325.

The pLI50-*tarG*-transformed S. aureus NCTC8325 was cultured at 37°C in 400 ml tryptic soy broth (TSB) culture medium overnight, supplemented with 17 μg ml^−1^ chloramphenicol. Then, the cells were collected by centrifugation; homogenized in buffer containing 50 mM Tris-HCl (pH 7.5), 500 mM NaCl, and 2 mM dithiothreitol plus 10% glycerol; and stored at −80°C if not for immediate use.

The cells were disrupted with a Constant cell disruption system (Constant Systems) with one pass at 30 kpsi. Cell debris was removed by centrifugation at 12,000 × *g* for 30 min. The supernatant was collected and incubated with the anti-Flag M2 affinity gel (Sigma) on ice for 1 h. Then, the resin was loaded onto the column and washed with 5 ml of wash buffer containing 50 mM Tris-HCl (pH 7.5), 500 mM NaCl, 10% (wt/wt) glycerol, and 0.04% (wt/wt) DDM. Protein was eluted with 6 ml of wash buffer plus 200 μg ml^−1^ Flag peptide. Subsequently, the protein was used for Western blotting assay. Flag tag antibody (catalog number 66008-3-Ig) was purchased from Proteintech.

### Extraction and quantification of S. aureus WTA.

Each portion of WTA was extracted from 10 ml of the S. aureus NCTC8325 strain cultured overnight. The extraction method strictly followed the protocol of the work of Covas et al. (45). The quantification of WTA was applied by the measure of the phosphate group from WTA using the ATPase colorimetric assay kit (Innova Biosciences) in 96-well plates at OD_650_.

### Data availability.

The accession number of our structure in PDB is 6JBH and in EMDB is EMD-9790.
