# Supplementary figures and images for: Cryo-electron Microscopy Structure and Transport Mechanism of a Wall Teichoic Acid ABC Transporter
Source: mBio. 2020 Mar 17;11(2):e02749-19. doi: 10.1128/mBio.02749-19 (PMC7078476; doi:10.1128/mBio.02749-19)

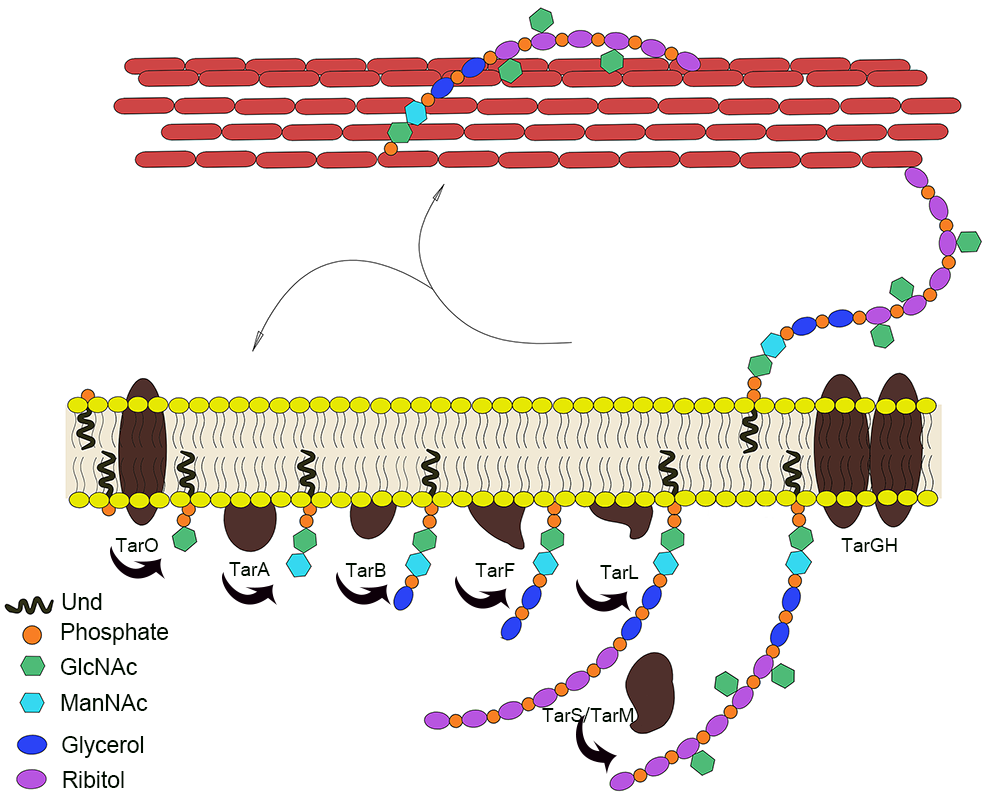

Supplement: FIG S1 [file mBio.02749-19-sf001.tif]

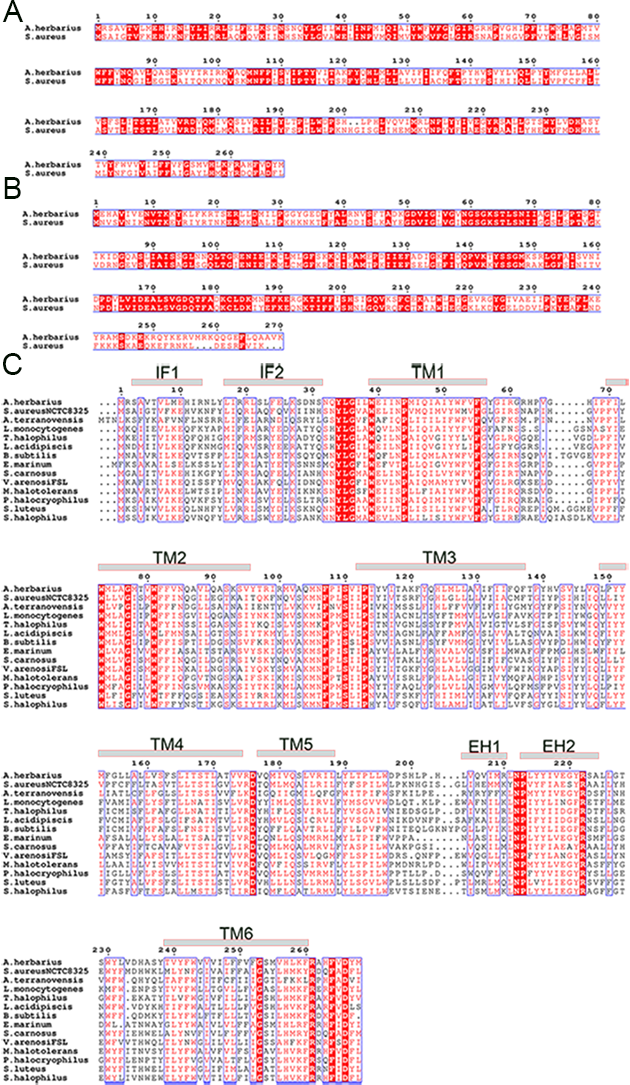

Supplement: FIG S2 [file mBio.02749-19-sf002.tif]

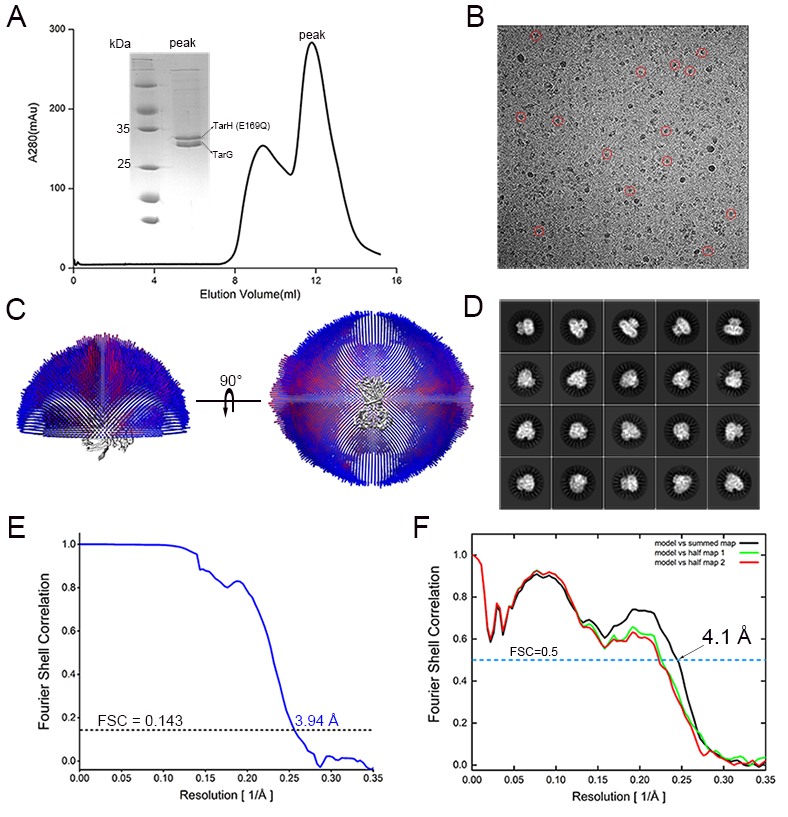

Supplement: FIG S3 [file mBio.02749-19-sf003.tif]

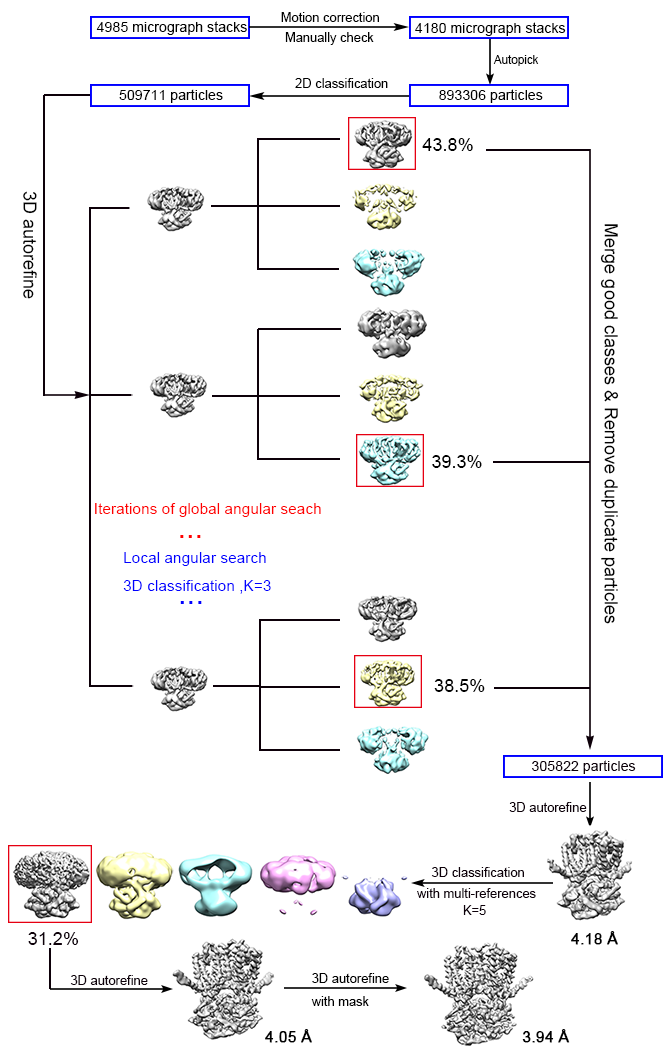

Supplement: FIG S4 [file mBio.02749-19-sf004.tif]

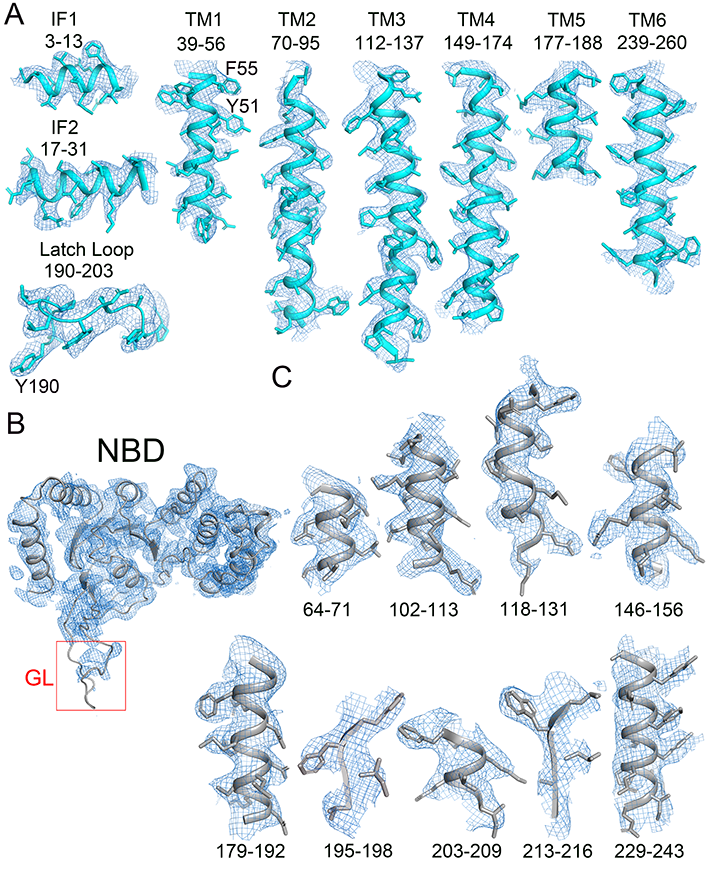

Supplement: FIG S5 [file mBio.02749-19-sf005.tif]

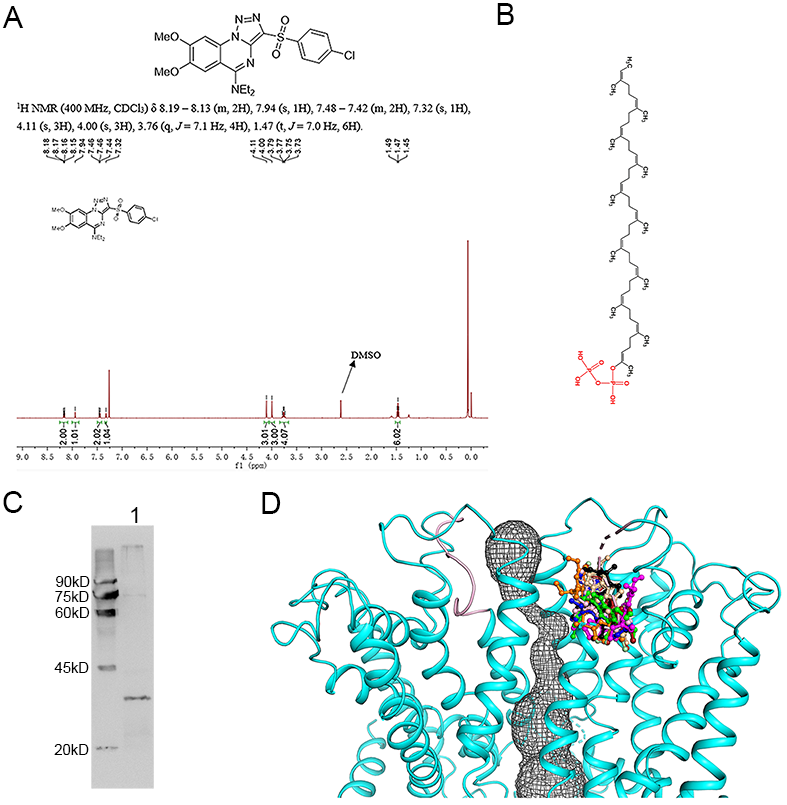

Supplement: FIG S6 [file mBio.02749-19-sf006.tif]
