# Supplementary material for: Cryo-electron Microscopy Structure and Transport Mechanism of a Wall Teichoic Acid ABC Transporter
Source: mBio. 2020 Mar 17;11(2):e02749-19. doi: 10.1128/mBio.02749-19 (PMC7078476; doi:10.1128/mBio.02749-19)
Supplement: TABLE S1 [file mBio.02749-19-st001.docx]

**Table S1.** **Cryo-EM data collection, refinement and validation statistics.**

|  | TarGH  (EMD-9790)  (PDB 6JBH) |
| --- | --- |
| **Data collection and processing** |  |
| Magnification | 59,000 |
| Voltage (kV) | 300 |
| Electron exposure (e^–^/Å^2^) | 64 |
| Defocus range (μm) | -1.5~-2.5 |
| Pixel size (Å) | 1.36 |
| Symmetry imposed | C2 |
| Initial particle images (no.) | 893,306 |
| Final particle images (no.) | 95,416 |
| Map resolution (Å)  FSC threshold | 3.94  0.143 |
| Map resolution range (Å) | 2.72-999 |
| **Refinement** |  |
| Initial model used (PDB code) | Ab initio model |
| Model resolution (Å)  FSC threshold | 3.94  0.143 |
| Model resolution range (Å) | 2.72-999 |
| Map sharpening *B* factor (Å^2^) | -198.567 |
| Model composition  Non-hydrogen atoms  Protein residues  Ligands | 8952  1062  0 |
| Bond lengths (Å)  Bond angles (°) | 0.007  1.214 |
| Planarity mean RMSD (°) | 0.007 |
| Dihedral mean RMSD (°) | 15.315 |
| Chirality mean RMSD | 0.066 |
| **Validation**  MolProbity score  Clashscore  Rotamer outliers (%) | 1.93  5.35  0 |
| Minimum non-bonded distance (Å) | 2.254 |
| Ramachandran plot  Favored (%)  Allowed (%)  Disallowed (%) | 85.48  14.33  0.19 |
| Map/Model correlation coefficients |  |
| CC (mask) | 0.81 |
